# Engaging girls with disabilities through cellphilming: Reflections on participatory visual research as a means of countering violence in the Global South

**DOI:** 10.1177/09075682211020067

**Published:** 2021-07-27

**Authors:** Xuan Thuy Nguyen, Marnina Gonick, Tuyen Bui

**Affiliations:** Carleton University, Canada; Mount St Vincent University, Canada; University of Illinois at Urbana-Champaign, USA

**Keywords:** Cellphilming, empowerment, Girls with disabilities, participatory visual methodologies, Vietnam, violence

## Abstract

Despite the fact that most countries have ratified the United Nations’ Convention on the Rights of the Child, girls with disabilities experience multiple forms of violence and oppression. Using a critical approach to arts-based research, we argue that while disabled girls experience multiple vulnerabilities due to the structural conditions which exposed them to violence, the use of participatory visual methods allowed them to reframe their stories. This article discusses the implications for reconceptualizing diverse childhoods in relation to methodologies for empowering disabled girls.

*“It’s urgent to remember that within feminist social movements and those fighting for the rights of people with disabilities, adults form the starting point for demands, and in this sense, take part in the erasure of girls with disabilities.”*

DAWN Canada (2020: 28).

## Introduction: Girls with disabilities and political activism

We begin this article by citing *Girls without Barriers: an intersectional feminist analysis of girls and young women with disabilities in Canada*, the most recent report conducted by the Disabled Women’s Network of Canada (DAWN), to highlight the invisibility of girls with disabilities within different feminist movements (DAWN Canada, 2020). The report recognizes multiple forms of oppression that girls with disabilities face at the intersections of class, race, gender, sexuality, and age. It acknowledges that “children, girls and young women offer a perspective on society and human relations that is important to take into account” and that “we recognize the oppressive relationship between adults and children” (p. 17). While feminist movements have demanded more inclusive recognition of difference, the experiences of girls with disabilities remain an under-represented issue. Clearly, then, there is a need to rethink the intersections between and among these dimensions of power which exclude and marginalize girls with disabilities at the intersection of multiple disadvantages.

In the context of the Global South, the exclusion and marginalization of girls with disabilities manifests at multiple levels. Despite the institutionalization of “children with disabilities” within the United Nations Convention on the Rights of the Child (UNCRC) (United Nations, 1989) and the Convention on the Rights of Persons with Disabilities (UNCRPD) (United Nations, 2006), girls with disabilities in the global South, along with the epistemic foundations upon which Southern knowledges are framed, have been largely sidelined in childhood studies. As Pérez et al. (2017) observe: “Whether in our writing or research collaboratives, when global south peoples have been invited to the table, at times, it’s been with the stipulation of theorizing or giving credit/dominance to global north critical theorists” (p. 80). The authors call for a “decolonial shift” (Mignolo and Walsh, 2018: 24) from the Eurocentric way of thinking about childhood in the global North by “centraliz[ing] global south onto-epistemologies” (Pérez et al., 2017: 80) and building connections between the global North and South in ways that engage with women and children of color and the violence they experience.

Furthermore, both feminist studies and disability studies have, historically, excluded girls with disabilities through the use of adult-centric approaches, where young people’s voices are often sidelined or omitted from research and feminist/disability organizing. Griffiths (2019) observes that young people are usually marginalized and experience difficulty in accessing disability politics. They are seen as naïve in various discourses surrounding disability organizing and movements. From a feminist disability standpoint, Ghai (2002) critiques the failure of the feminist movement in centering disability oppressions as one among many dimensions of oppression against which the movement is fighting. She asks: “What now can be done about feminists’ inattention to disabled girls and women, and how would feminist discursive attention look and read if, initially, they had been involved in its development?” (p. 62)

Engaging with the voices, representations, and experiences of girls with disabilities in the global South, thus, is crucial for critical childhood studies because it helps to decolonize the normative framings of childhood, disability, and gender, and instead, opens up new spaces for questioning the politics of research: who conducts research, in whose interest, who benefits from research, and how, given their absence from childhood studies, girls with disabilities in the global South can represent themselves through research productions (Nguyen, 2016, 2020). We believe, then, that it is urgent that the voices of girls with disabilities in the global South be heard as a way of tackling social injustice in the Global South and transnationally.

In this article, we examine how girls with disabilities in Vietnam shape their stories in response to disability, gender-based, and racialized forms of violence. We use a critical approach to arts-based research to refer to “the notion of possibility, the *what might be*, of a research tradition that is post-colonial, pluralistic, ethical, and transformative in positive ways” (Finley, 2018: 561). Drawing on feminist disability studies, we define disability as a socio-political construct that reflects how difference is discursively and materially produced by colonial, imperialist, and capitalist forces, and how intersecting forms of oppression and disablement produced by transnational capitalism have devalued and excluded disabled, racialized, and gender non-conforming bodies (Erevelles, 2011). Within this critical frame, then, disability is “not a physical defect inherent in bodies but rather a way of interpreting human variation” (Garland-Thomson, 2018: 12). We utilize post-colonial and feminist disability studies to shed light on the temporal and relational aspects of disability, vulnerability, and embodied experiences (Kim, 2017) while highlighting the importance of agency and materiality in framing the relationships between body and the world (Garland-Thomson, 2011). In so doing, we hope to counter the dominant perspective of “girls with disabilities” that are often portrayed in international development discourses.

We will begin by revisiting the discourses of “girls with disabilities” in international development and academic research. We will then discuss the challenges of such discourses in failing to recognize the diversity of girls with disabilities in the global South. Next, we will discuss the use of participatory visual methodologies (PVM) within our project, Transforming Disability Knowledge, Research, and Activism (TDKRA) to illustrate the ways in which the disabled girls we worked with used PVM to counter dominant narratives and to set the stage for their activism. Using interviews we analyze multiple forms of violence that disabled girls experienced. We demonstrate how PVM can empower disabled girls to express themselves while resisting oppressive forms of violence in their schools, families, and communities.

Drawing on our earlier work on girls with disabilities in the global South (Nguyen, 2016, 2019; Nguyen and Mitchell, 2014; Nguyen et al., 2019), we use an intersectional perspective which is defined as “an analytic lens [that] highlights the multiple nature of individual identities and how varying combinations of class, gender, race, sexuality, and citizenship categories differentially position each individual” (Collins and Bilge, 2016: 8). It is imperative for understanding the multiple forms of violence experienced by girls with disabilities in the global South and for analyzing the various strategies girls use to resist the forms of oppression shaping their lives.

## Who are “girls with disabilities”? Contested discourses in academic/activist spaces

To examine the ways in which girls with disabilities are represented in both academic literature and in documents published within global organizations, we draw on Foucault’s idea that discourses do not merely describe existing social categories, they create them (Foucault, 1969). In other words, we come to think about and understand girls with disabilities through the discourses (or their absence) that circulate about them in texts produced in contexts such as these. The term “girls with disabilities” is situated within contested power relations, which results in disabled children and young women navigating a complex and contradictory set of discourses with which to negotiate their identities. On one level, there is increasing global attention to the issue of disability, by organizations such as the United Nations, but this attention rarely addresses differences such as gender, race, caste, class, or region. On another level, local hierarchical systems of domination based on the structural maintenance of sexism, classism, and ableism in a range of institutions (schools, churches, and families, among others) continues unabated.

With the passing of human rights accords and development charters such as the Millennium and Sustainable Development Goals, and Education for All Frameworks, gender equality and disability rights are diffused through global agencies such as the World Health Organization, UNESCO, USAID, and UNICEF (Meekosha, 2008; Swartz and Marchetti-Mercer, 2018; World Health Organization, 2011). The impetus for women and girls and disability rights is thus not always a bottom-up demand, attentive to the most marginalized, but a top-down incentive for improving international relations and increasing donor aid. Don et al. (2015), Milligan (2014), Nguyen et al. (2015), Russell (2016), Ramaahlo et al. (2018), and Vavrus (2003) note how in Rwanda, Tanzania, India, Kenya, Vietnam, Iran, and other parts of the Global South, gender equality and disability rights at times become strategies for “doing” economic development from “outside in,” rather than transforming the social and cultural structures of society from the “inside out.” This development approach is enmeshed within colonial legacies, where the mainstreaming of women and girls with disabilities are seen as a pre-condition for neo-liberal economic development (Dingo, 2007). The contested power relations exist between high-level national and international policy demands and incentives for the human rights of disabled women and girls, and the local governments which directly shape their complex experiences in the South. In many instances, development discourse embedded within a neo-colonial approach to inclusion marks marginalized women and girls with disabilities as invisible (Nguyen, 2015).

Official definitions of childhood violence as framed within the UNCRC and mainstream child protection organizations often fail to grasp the particularities of disabled children’s experiences which often go beyond those experienced by non-disabled children. Hernon et al.’s (2015) review reveals how disabled children are largely excluded from mainstream child protection policies, while their “voices” on issues of violence and support are absent from research (Stalker and McArthur, 2012). Moreover, as Nguyen et al. (2015) point out within the legal provisions for “Children with Disabilities” in Article 23 of UNCRC, specific reference to girls with disabilities is entirely absent (United Nations, 1989). They argue further that there has been a lack of attention specifically to disabled girls’ experiences: their stories have been absent in dominant narratives of disability rights (Nguyen et al., 2015: 775).

## Conducting participatory arts-based research with girls with disabilities in Vietnam

In its long-standing history, Vietnam embodies competing ideologies on girls and women with disabilities. Specifically, as a post-colonial and socialist country, Vietnam promotes gender equality. Its official discourses such as the *Law on Gender Equality* highlights equality between men and women and prohibits gender discrimination in all forms (Government of Vietnam, 2006, Law 73/2006/QH11). However, there is no specific mention of disability in this law. Interestingly, despite UNCRPD’s incorporation of intersectionality (United Nations, 2006), the state passed the Law on Persons with Disabilities in 2010 after having signed the CRPD with minimal mention of gender. There are two specific mentions of gender in relation to health care for pregnant women and social protection such as hygienic support for menstruation, and yet, there is no specific mention of girls with disabilities in this law (Government of Vietnam, 2010). The amended *Law on Children* includes disabled children within a specially designated group called “children in special circumstances” (Government of Vietnam, 2016, Law No. 102/2016/QH13: Article 10). The law states that these children “are supported, cared for, educated in special education services to rehabilitate their function, develop independent living skills, and integrate into society” (Government of Vietnam, 2016, Law No. 102/2016/QH13: Article 35). The law uses a functionalist approach to disability, as indicated in its language on rehabilitating their function, suggesting a need to fix the disability before disabled children can be into schools. Clearly, then, while the state has promoted human rights for women, children, and disabled people, girls with disabilities are invisible across these legal domains. They are not recognized by the state as a group whose rights have been left out of state protection.

The TDKRA project addresses the absence of knowledge about girls with disabilities in the global South. It uses participatory visual methodologies (PVM) as an arts-based approach along with traditional methods such as in-depth interviews and focus groups. In partnerships with three Disabled People’s Organizations, we also sought to tackle the boundaries between research and activism with women and girls with disabilities in Vietnam. In earlier research with girls with disabilities in Vietnam, we found that PVM is an important tool for engaging girls with disabilities to express themselves and to re-frame issues from their own perspectives (Nguyen, 2016, 2020; Nguyen et al., 2015). Therefore, in the TDKRA project, we worked with girls and women with disabilities in Vietnam using different PMV methods such as drawing, cellphilming, and photography to engage them in co-producing knowledge and fostering forms of activism.

Our project was designed in response to families’ and communities’ concerns about the inclusion of girls with disabilities into education and society. The local fieldwork included training women with disabilities to do data collection. In each community, these women received 5 days of training in interview skills and participatory visual methods by team members, including our research assistants with disabilities. This was followed by their involvement in working with disabled girls in their communities. In our first year of fieldwork, the girls worked in small groups of 4–5 girls facilitated by the older women to make cellphilms—short videos made with cell phones. We provided basic instructions on the process including selecting a topic, creating a storyboard, and simple cell phone filming techniques.

The research team developed focus groups and interview guides through consultations with the DPO partners. The interview questions were adapted by the women in the training to ensure the cultural relevance and appropriateness of these materials to the local contexts. In some instances, we took advice from the local DPOs in workshop organization and community engagement events, ensuring that our research practices were culturally relevant and responsive. The research team, including research assistants with disabilities who are members of these communities, participated in the data analysis and the writing of the research report.

## Participants and settings

The TDKRA project was implemented in three disadvantaged communities of Vietnam including Bac Tu Liem (Hanoi), A Luoi (Hue), and Ninh Kieu/Binh Thuy (Can Tho). We selected these communities because they are distinct in terms of their socio-economic, ethnic, and political locations. The communities in Bac Tu Liem, and Ninh Kieu represent emerging urban areas with diverse socio-economic conditions, while A Luoi is a highly disadvantaged ethnic minority^1^ community, heavily affected by Agent Orange—one of the most toxic herbicides used by the American military during the Vietnam War. There is a high rate of impairment for second and third generations of children in A Luoi whose family members were exposed to the herbicide during the War (Ngo et al., 2013). These include blindness, physical, and intellectual disabilities, as well as multiple forms of illness such as cancers and brain tumors.^2^

We approached Disabled People’s Organizations (DPOs) to recruit participants from the three communities. The result is 86 participants with disabilities (55 girls and 31 women). Criteria for inclusion and exclusion include: (a) aged 10–18 years old; (b) having experienced some forms of discrimination in and by the educational system; (c) having one or more disadvantages associated with their disability, class, gender, and ethnicity; (d) experience of barriers to education and community, and have potential leadership for educational rights. The DPOs used the recruitment procedures and criteria to recruit participants in their local communities and consulted with the team when questions arose.^3^

Some participants had prior relationships with the DPOs, which we view as a positive since one of the key objectives of the project is to build and support relationships between the girls, women and the DPOs to further their activism for inclusion. We also recognize that this means that girls and women who were not already connected to DPOs may not have had access to the project.

In this article, we draw on the cellphilms produced by the girls and interview data conducted by the trained women with disabilities. The visual methods were introduced in all three sites under the guidance of the researchers. In the case of cellphilming, some local women had been trained with filming and video-editing skills prior to their participation in this project. The making and presenting of the cellphilms created much excitement for the girls and were much more successful for participant engagement than the focus groups and interviews.^4^

## Findings

### Violence against girls with disabilities: An intersectional perspective

Violence against girls with disabilities is complex. Many girls identified non-disabled school peers as the perpetuators of hitting and teasing. For example, as an ethnic girl with disabilities, Ut (16 years old) said that her classmates often made fun of her: “Whose child is it? Still going to school with a disability?” When asked why they acted that way, she said: “Because they hate me, hate my disability.” Another girl, Tri Tue, shared the experience of being bullied by a boy in class. “He thought I am a person with disabilities, so he teased me.” Being isolated and hit by her peers, she got angry and hit them back. These students then made fun of her disability: “You are a person with disabilities! Do you dare hit me?”

These comments demonstrate an ableist culture where the presence of disability is seen as unacceptable. The girls reported being hit, beaten, insulted, assaulted, attacked, pushed, and slapped on their face and bodies. They were hit on the head and back, pinched on the face, their feet were stepped on, and they were pushed until they fell down. Some girls had their hair pulled and stones thrown at them. In some cases, a group of non-disabled peers collectively attacked the girls because of their disabilities. A girl who chose the pseudonym Linh shared experiences of being physically and verbally abused by “adults,” who appeared to be older students at school:

Linh: The adults despised me!

Facilitator: The adults despised you? How were you despised?

Linh: They spoke badly.

Facilitator: What did they say about you?

Linh: “A person with a birth defect,” sometimes they say “A person with a disability.”

Facilitator: But who are the adults?

Linh: Many, I do not know their names. They did it when I went to school.

The “adults” are not defined by age but by their capacity to bully younger students. She described these adults as bullies because they “spoke badly” of her disability. At the same time, her experience with violence was not restricted to school. She said that she was occasionally hit by her father with a broom: “the broom was broken, but I was still OK!” (Interview with Linh, 2018).

Another girl with a hearing impairment said that she was hit by her parents when asked to do some household chores but she did not hear the instructions. She said her parents “forgot” that she had an impairment. Trai Tim, an autistic girl living with her father and stepmother, was hit multiple times by her father and sibling due to what she described as “naughtiness.” In the conversation, Trai Tim expressed her pain and powerlessness:

Trái Tim: I think God hates me!

WWD3: No.

Trái Tim: I kept praying but it never came true.

WWD3: What did you pray for?

Trái Tim: I prayed no one would scold me

WWD3: You should tell everyone to stop scolding you.

Trái Tim: I kept praying but it never came true.

(Interview with Trai Tim, Hanoi, 2018)

Trai Tim also shared her painful experience when her brother used an electric wire to hit her: “He hit me with a broken charger cable” (Interview with Trai Tim, 2018). Another girl stated that she was scolded by her father whenever she did anything wrong. It made her want to end her life: “I feel unhappy when living a . . . life, I just want to die” (Interview with Mot, 2018).

The lack of decision-making power in relation to their bodies can be seen as a structural problem facing disabled girls in their families. Due to her epilepsy, for example, That Tha dropped out of school in grade two and has since stayed at home. She lives with her single mother who does causal jobs and was often away from home. Located in a very remote area in the suburb of Hanoi City, That Tha was totally marginalized. She had a sexual relationship with a young man she met on-line and got pregnant. The idea to have the child seemed not to be hers, but her mother’s. Her mother advised her to “ask him for a child so that you can have someone to rely on” (Follow-up interview with That Tha, 2020). While her mother’s suggestion may have been made out of concern for her daughter’s future, there is an implicit assumption that disabled girls cannot take care of themselves independently. In this case, parental power intersects with institutional ableism, sexism, and classism in creating multiple axes of marginalization.

In other instances, we found that ethnic minority girls faced higher level of discrimination than the majority group (Nguyen et al., 2019). In public schools in the rural area of A Luoi, ethnic minority girls reported being despised, looked down upon, and teased by Kinh (the majority) students. Being an ethnic minority girl and having a disability could make one a target of violence. For example, Kinh students viewed them as stupid, since their mother language was not Vietnamese. One girl recalled being told, “What an ethnic minority bitch!” A participant exposed to Agent Orange shared her experience: “They used to threaten, mess up our hair and looked down on ethnic people. They teased us for wearing tattered clothes to school” (Focus group in A Luoi, 2018). Thus, here we have examples of violence produced through the disabled girls’ experiences with institutional racism and disablism.

### Taking action: Girls with disabilities picturing violence

The use of participatory arts-based research was designed to allow the participants to respond to violence in creative and playful ways. It shows how disabled girls agentically engaged in action in response to intersectional oppressions. The prompt we offered, “explore an issue or concern in the lives of girls with disabilities,” is a relatively open prompt allowing for each group’s interpretation. The groups chose a theme that was most relevant and poignant to them. After creating the storyboard, the groups assigned roles to each of their team members. They practiced their scenarios and filmed themselves, watching their finished video in their groups. Then all groups were brought together to view and respond to the films. We collaborated with the DPOs in each community to screen the films to parents, teachers, educational administrators, and community leaders.

One 2-minute film tells a story of school bullying. Titled, *Paying a Price* the film shows a boy and a group of girls playing in the school ground. An older boy bullies a girl with visual impairment calling her “four eyes.” The girl responds by refusing to speak to the bully when he tries to engage her. In the last scene, the boy recognizes his bullying and apologizes to the girl and her friends. The film ends with the boy and the girls holding hands in the playground as a way of re-connecting. The film sends a simple message about disabled girls’ ability to determine the terms of engagement with others. Her refusal and then forgiveness after amends are made suggests a form of agency over herself and others.

A 3-minute cellphilm, produced by a group of girls and young women in A Luoi, is focused on violence due to disability and ethnicity. The film begins with a learning activity in a visual art class. The teacher asks the students to comment on the drawings they have completed. An ethnic girl with disabilities tries to share her opinion. A student, points with a ruler at the girl: “You must not have your opinion!” The camera then shows the ethnic girl with lowered face, illustrating her feeling of powerlessness. In another scene in the classroom, the camera shows another student threatening to exclude the minority girl: “Get out of here so that we can discuss!” We then see the class dismissed and the ethnic girl remaining in the classroom to pick up garbage. In the final scene, the violence continues. The main character is seen serving her presumably non-disabled (and ethnic majority) peers. The girl is asked to serve a drink and to clean the cup and refuses to do so. The bully slams her hand on the table, pointing her finger at the girl: “If you refuse to follow my instructions, I will beat you! Did you know that?” While the film is untitled, the participants sent a clear message about equality and non-discrimination with a poster held up at the end of the film: “Treat us like ‘non-disabled’ people!”

It should be noted that the participants had never acted before. Some had never touched a camera or cellphone in their lives. What arts-based approaches offer however, beyond these opportunities is, access to ways of knowing and routes to knowledge that include but are not limited by existing language and narratives (Gonick, 2017). As Gonick (2017) outlines, for participants who may have had no experience of sharing personal stories, have no habit of self-reflexivity, or are not fully conscious of the forces at work shaping their lives, arts-based approaches may offer a means of re-imagining what might be. One group discussed their impressions of their films:

Mickey: I like the video.

Facilitator: why do you like the video?

Mickey: it is meaningful.

Facilitator: how is it meaningful?

Mickey: it means—don’t look down on persons with disabilities.

## Discussion

This article shows that PVM provides an opportunity for participants with disabilities and other forms of marginalization to speak back to dominant narratives. Their narratives challenge the absence of girls with disabilities in discourses about disability, development, gender, and education in the global South. Sometimes PVM is framed as offering empowerment, voice, and/or agency to participants (Gonick, 2017). However, as Gonick suggests, even with this shift in the research dynamic, it is important not to romanticize relations between researchers and researched. As feminist researchers have argued for decades, there are always unequal power relations shaped by differences such as age, race, gender, class disability, and sexuality, and these need to be engaged with, acknowledged and theorized by researchers.

As a group of adult researchers, we acknowledge our unequal power relationships with girls with disabilities and the communities with whom we are engaging in this project. The first and third authors grew up in Vietnam, working with children and youth with disabilities while the second researcher experienced Vietnam for the first time with this project. Our attempts to shift this structure of power by using PVM does not mean that we did not hold certain forms of power as adult researchers. In fact, it requires us to engage in “critical reflexivity” (Strega and Brown, 2015) by acknowledging our positionalities and values as well as “the political struggles that we have not necessarily resolved” (p. 10). We must ask, for instance, how are we positioned to interpret the stories framed by girls with disabilities in the global South? How can adult researchers from the global North support these girls to re-story their experiences without overpowering them in our politics of interpretation?

This article has engaged with the local dimensions of experience of girls with disabilities in the global South. Within a global context disability rights and gender mainstreaming have been mobilized through legal reforms. Despite these transnational movements, disabled girls continue to represent a sharp contrast to the socialist regime, where the core values and structure of institutions in relation to the labor market have perpetuated the perception that these girls are an exception to the ableist nation-state (Nguyen and Mitchell, 2014). As Vietnam’s ideological shift into a neoliberal economy since the late 1980s has reframed its vision of entrepreneurial citizenship capable of participating into the market (Nguyen, 2015), disabled girls are “included” as long as they can re-capacitate themselves as the neo-liberal subject. And yet, as Todd (2016) argues, this capacitated ideology of girlhood excludes disabled girls, who are seen as incapable of fitting into neoliberal norms.

Neoliberalism has subjected young women and girls with disabilities to exclusion. On one hand, these young people have remained invisible within existing laws on children. On the other hand, the “inclusion” of disabled girls and women into the competitive neoliberal market has perpetuated a system of violence which marks them as excludable from the economy (Nguyen, 2015; Stienstra and Nguyen, 2020). Despite the fact that Vietnam ratified the UNCRC and enacted the amended *Law on Children*, the law does not recognize diverse experiences of childhood and children. By constructing children as vulnerable and in need of protection, however, this law has left untouched the structural and intersectional conditions that create and perpetuate inequalities against multiply marginalized children.

Furthermore, while existing laws have promoted gender equality (Government of Vietnam, 2006), such laws have not recognized the ways in which disabled women and girls experience gender discrimination differently. For example, there is no recognition of disability and childhood with the *Gender Equality* law. In contrast, the majority of girls and young women with disabilities in this study revealed that they felt that they experienced inequality because of their disabilities. While their perceptions on disability have shifted through their involvement in TDKRA (Nguyen and Stienstra, 2020), we have shown that girls with disabilities are more likely to experience structural, verbal, emotional, and physical violence because of the intersections between institutional classism, sexism, racism, and ableism that played out in their families and schools. Clearly, the lack of understanding of intersectionality among disability, gender, ethnicity, and childhood identities in relation to ableist, sexist, racist, and adultist ideologies in Vietnamese laws, cultures, and society has perpetuated disabled girls and women’s invisibility and increased their vulnerability.

At the same time, the ways in which disabled girls in Vietnam engaged in the TDKRA project reflects their capacity to picture various aspects of violence from their own ways of seeing. This process reflects their capacity to claim agency through consciousness-raising activities such as cellphilming to picture alternatives. With basic filming skills and techniques, they exposed different forms of discrimination and violence, illuminating different aspects of unequal power relations which they endure in their everyday lives. As demonstrated by their cellphilms, the girls were well aware of the structures of inequalities. While not aiming to picture the “truth” of disabled girls’ lives, this participatory process enabled disabled girls to engage in the process of meaning-making. As Gonick et al. (2014) note, “The methodological question is not whether or not truth is found, but addresses the multiplicity of truths that are produced and through what technologies” (p. 44). Furthermore, the participants not only pointed out the problems but also suggested solutions.

## Conclusions: Implications for childhood studies

While childhood studies has been increasingly interested in the lives of marginalized young people, the attention has mainly been focused on the Global North. Studies of girls and childhood more broadly in Global South contexts remain scarce (Kirk et al., 2010). Questions also remain within the field about the creation and implementation of research methodologies that are capable of representing the complexities of issues such as ethics, decolonization, tokenism, and privileging or romanticizing the voices of participants in the Global South. We suggest that the cellphilms made by the girls in our project, and PVM more broadly, can challenge norms of childhood directly and indirectly, with subtlety and with affective power. PVM, thus goes some way toward facilitating a more expansive field of childhood studies (Mitchell et al., 2016).

As art created by girls with disabilities in the Global South, the cellphilms create new discursive spaces for self-representations, visibility, and voice to replace the more common phenomenon of representations of young people made by adults, absence, and being spoken about by others. The cellphilms are vehicles for the girls to experiment with meaning making, competences, and the construction of worlds from their own perspectives as girls with disabilities.

The work challenges Childhood Studies on a number of fronts, posing questions such as: How does the inclusion of girls with disabilities from the Global South re-define norms and meanings of childhood? How does beginning from a place of supporting girls with disabilities as agentic subjects, expand our understandings of agency? And how does centering the interplay of age, cultural context, sex, and gender generate new ethical feelings about and responses to injustice? How do particular definitions of girlhood and childhood include/exclude disabled girls? Finally, how can disability challenge and speak back to neo-liberal constructions of girlhood?

As a field that frequently crosses disciplinary boundaries, childhood studies is perhaps an ideal site for resisting established power relations within the academy and for promoting non-totalizing epistemological values. Children’s rights, particularly those curtailed by patriarchy, ableism and lack of resources, as well as the structures that enable and restrict control over their lives could be focal point for the field. Children are often studied in consideration of the future adults they will become, rather than as individuals and citizens with a full set of rights and expectations in the present. The emphasis is especially true for girls, who are seen mainly in terms of their future roles as mothers and the nurturers and mainstays of families and communities. Images of “the child” in academic writing, in policy and in organizations need to better reflect the complexity of children’s lived experience across the array of disability, gender, sexuality, and ethnicity.

The cellphilms and the participatory processes through which they were created, suggest that much can be learnt from understanding the construction of girls living with disabilities in the global South and how our current limited exploration of this effects how society prevents, identifies and responds to violence and other abuse and associated trauma.

## Notes

1. Culturally, Vietnam is home to 54 officially recognized ethnic groups in a country of ninety-seven million people. Many ethnic minorities in Vietnam have experienced unequal power relations with the Kinh ethnic majority controlling the political and socio-economic lives of minorities (McElwee, 2008).

2. The high rate of impairment refers to incidence of individuals affected by Agent Orange and potential risks of being exposed to it. There is a lack of prevalence data on disability and impairment in Vietnam (Ngo et al., 2013). Additionally, available data tends to be unreliable and usually employs a medical models with disability, which is in contrast with a critical disability studies perspective which we adopts in this paper.

3. Due to specific local conditions, some DPOs requested expanding the age range of the girls from 18 to 20 to allow for access to some girls in their communities. We received approval from the university’s ethics board to expand the age range.

4. For other decolonizing and participatory approaches used in TDKRA, see Nguyen (2019, 2020); Stienstra and Nguyen (2020); and Nguyen et al. (2019).
